# Knowledge of Medical Education on Maternal and Child Primary-Care Among Physicians: A Cross-Sectional Study

**DOI:** 10.3389/ijph.2024.1606536

**Published:** 2024-07-04

**Authors:** Haribondhu Sarma, Pham Ngan Giang, Matthew Kelly, Tran Van Anh, Chalapati Rao, Nguyen Phuong Hoa

**Affiliations:** ^1^ The National Centre for Epidemiology and Population Health, Australian National University, Canberra, ACT, Australia; ^2^ Preventive Medicine Centre, Hanoi Medical University, Hanoi, Vietnam

**Keywords:** continuing medical education, commune health stations, maternal and child care, Vietnam, remote and mountainous areas

## Abstract

**Objectives:**

To assess the pre-training knowledge of Commune Health Stations (CHSs) physicians in Vietnam on pregnancy and child care.

**Methods:**

A cross-sectional study was conducted and a pre-training questionnaire was administered with physicians working at CHSs in three mountainous provinces of northern Vietnam. Calculated mean knowledge score and estimated adjusted odds ratios (AOR) to compare the relative odds of occurrence of the outcome “answering more than half of questions correct,” given exposure to the physicians’ characteristics.

**Results:**

A total of 302 CHS physicians participated. The mean number of correct answers across all participants was 5.4 out of 11. Female physicians are 2.20 (95% CI: 1.35–3.59, *p* = 0.002) times more likely to answer correctly than their male counterparts. Physicians aged 35 years or more were significantly less likely to answer correctly (AOR 0.35, 95% CI: 0.15–0.81, *p* = 0.014).

**Conclusion:**

The study found that participating physicians possessed relatively low knowledge of pregnancy and child care. The study also found significant disparities in this knowledge according to the physicians’ characteristics. Thus, it is recommended the requirement for continuing targeted medical education to improve doctors’ proficiency in these areas.

## Introduction

Vietnam has made vast improvements in maternal and child mortality in recent decades. Overall, the maternal mortality rate dropped to 54 per hundred thousand live births in 2014 from 139 in 1990, and under-five mortality declined to 22 per thousand live births in 2015, from 51 in 1990 [1]. Several factors have contributed to these improvements, including implementation of universal health coverage and a five-fold increase in *per capita* government health expenditure prioritizing essential maternal health interventions such as tetanus vaccination of pregnant women and children, skilled birth attendance, and emergency obstetric and newborn care services [1]. Despite this progress, healthcare delivery in remote, mountainous areas of Vietnam still lags far behind. In these disadvantaged areas, infant and child mortality rates are higher and life expectancies lower, and mothers are less likely to seek antenatal or postnatal care or to deliver in a health facility [2, 3]. A longitudinal analysis of Multiple Indicator Cluster Survey (MICS) data revealed higher under-5 mortality rates in the remote mountainous areas than in the Red River Delta regions [4]. Vietnam’s government has established Commune Health Stations (CHSs) at the community level, particularly in rural and remote areas. These stations act as the first contact points for access to the public healthcare system and have a role in providing effective health services for pregnant women, infants and young children. CHSs also supervise and monitor personnel charged with implementing health education initiatives in the communities [5].

CHSs number approximately 11,162 across the country and delivery primary care services to an average catchment population of 7,000 to 8,000 people each [6, 7]. CHSs are typically staffed by five to ten healthcare workers, depending on the size of the commune’s population, and are led by a trained medical doctor [8], generally a graduate in family medicine, with or without post-graduate speciality training [9]. Substantial increases in public financing in the last decade have aimed to improve primary care in Vietnam by developing infrastructure and improving staff quality at the community level [10, 11]. Studies conducted in primary care settings, including CHSs in northern Vietnam, observed high capacity for delivering preventive and curative services at the community level following these investments in public health [12, 13]. A recent assessment of health service quality supports those findings, as CHSs were found to be the preferred choice of study participants [10]. The same study also demonstrated that CHSs obtained significantly higher quality service scores than did other healthcare services in Vietnam.^10^ However, that study did not analyse CHS scores by location, and it is assumed that health service quality may be lower in remote areas, as these facilities often report limited capacity and resources. The types of health services dispensed are also key determinants of health service quality. Previous analysis has demonstrated that respondents suffering from non-communicable diseases were more likely to be satisfied with the CHS services compared to the patients in need of maternal or child healthcare [10]. In addition, qualitative research has revealed that Vietnamese mothers preferred private health facilities or district-level hospitals for antenatal care and delivery, as they perceived that better care and more diverse services were available in these facilities compared to their local CHSs [14].

Patients often eschew CHSs in favour of secondary- or tertiary-level health centres, expecting to receive better care from these higher-tier facilities [9]. This suggests that there may be opportunities to improve the quality of services in the CHSs. Research conducted with CHS physicians has highlighted the importance of ongoing training of health service providers for improving services in primary care settings [9, 11], and opportunities for doctors to refresh and expand their knowledge in the field were deemed to be particularly valuable. A recent policy paper also underscores the need for promoting scientific research to develop skilled health human resources, such as by reforming training and retraining strategies for medical staff in the disadvantaged provinces of the northern and central regions [15]. Policies have also emphasised increasing the numbers of medical staff with high levels of clinical competence to meet patients’ expectations and increase satisfaction [15].

Continuous knowledge enhancement initiatives for the physicians working in the CHSs are critical to improving the health service quality. As part of national initiatives for health human resources development, Vietnam’s government enacted a Master Plan for Health Workforce Development (MPHWD) from 2012 to 2020, intended to increase the quality of care at the community level through training and education of existing staff at the CHSs [16]. Despite such initiatives, there are still frequent reports that current approaches for human resource development and training in CHSs are not adequate [17–19]. Additionally, staff in the remote areas had limited access to training compared to their urban counterparts [12]. In 2020, the MPHWD was renewed for ten more years (2020–2030). Recurrent training activities, including short courses on day-to-day clinical management, as well as graduate and post-graduate education focusing on primary healthcare, have been proposed and may enable providers at CHSs to acquire cutting-edge knowledge that would strengthen their skills and abilities [20].

In 2019, the Hanoi Medical University (HMU) was commissioned to develop a training curriculum covering a range of primary healthcare topics, including maternal and child health. The HMU piloted and tested these training activities in three mountainous and remote Northern provinces of Vietnam. Lessons drawn from this training program are expected to help advance the national training strategy for CHS doctors and identify and address the potential training needs of these physicians. Data from these pilot studies may also help guide the integration of such training courses into existing medical education programs across the country. This paper assessed the pre-training knowledge of Medical Education on Maternal and child among Primary-care Physicians in Vietnam.

## Methods

### Study Design, Setting and Population

A descriptive cross-sectional study was conducted in 2019 with general practitioners (physicians) working at the CHSs in three remote, mountainous provinces of northern Vietnam: Ha Giang, Yen Bai and Dien Bien. The study participants were physicians attending a training program organized by the HMU as part of a Continuing Medical Education (CME) initiative in Vietnam. The total populations of the study provinces were 854,679 in Ha Giang; 821,030 in Yen Bai; and 598,856 in Dien Bien, respectively. The provinces were served by a total of 426 physicians working in 487 CHSs. Of these, 302 communes have doctors working at health stations, and all these doctors (100%) received CME training and participated in this study. The remaining communes do not have doctors, only assistant doctors who are trained in other courses. As part of CME programs, the HMU organized eight training sessions during 2019. Before the commencement of the actual training sessions, a pre-test questionnaire was administered by the HMU to all physicians participating in the program.

### Study Materials

For assessing pre-training knowledge of participant physicians, at first, structured questionnaire was drafted, then validated the questionnaire through a pretesting of the questionnaire with a sample of 20 doctors. With the feedback from pre-test participants, the questionnaire was revised to improve the content, style and logical flow. The overall questionnaire comprised a general component with questions on the basic tasks and responsibilities of CHS physicians in maternal and child care. The three main sections of the final questionnaire consisted of basic socio-demographic information questions (i.e., age, gender, and work experience), questions on the basic tasks and ideal roles of CHS physicians, and questions related to clinical practice. The clinical component of the questionnaire contained three parts: 1) common medical emergencies, 2) maternal and child care and 3) non-communicable disease management. The questionnaires were presented in multiple-choice format with doctors asked to choose the most appropriate response(s). The correct answers for each question were assigned based on the “Instructions for examination and treatment of common diseases,” outlined by the Medical Examination and Treatment Administration of the Vietnam Ministry of Health.

This paper analyses data from the section on maternal and child care, which contained 11 questions. The questions and response options are detailed in Supplementary Table S1. Six questions pertained to maternal health, assessing doctors’ knowledge of critical pregnancy care, including standard protocols for antenatal care and pregnancy-related danger signs. Five questions assessed physicians’ basic knowledge of child care with questions covering topics such as the diagnosis and management of dehydration in childhood diarrhoea, the IMCI protocol, and danger signs requiring prompt referral of sick children to the Provincial level or Central level health facilities (Supplementary Table S1).

### Data Collection

Printed questionnaires and written consent forms were distributed to the participants, who were instructed to independently provide responses to the questions and given 15 min to complete the questionnaires. The questionnaire was self-administered by the participants, however, faculty members of the training program supervised the pre-test sessions. The same procedures were applied for collecting data from all eight training sessions.

### Data Analysis

First, descriptive statistics were calculated to characterize the participating physicians by gender, age, seniority, and province of practice. A comparative analysis of test performance for each of the descriptive variables was also performed, categorised by sex (male, female), age (<35 years, ≥35 years), seniority (years of working as CHS physicians, <10 years, ≥10 years), and location (province). In the second step, the proportion of physicians who correctly answered each of the 11 questions on maternal and child care was measured, then the questions were categorised into two groups (pregnancy care and child care). The mean number of correct answers for each physician was calculated, and these mean scores were compared between the groups for each of the physicians’ characteristics described above. T-tests were used to determine the significance of comparisons based on age, gender, and seniority. To assess statistically significant differences in mean scores between the three provinces, one-way ANOVA was used. In the third step, the generalised estimating equation (GEE) model was applied, and crude and adjusted odds ratios (OR) were estimated to compare the relative odds of the outcome “answering more than half of the questions correctly,” given exposure to the variables of age, gender, seniority, and province. Three GEE models were performed: one for all questions, one for questions on pregnancy care, and one for questions on child care. Pearson’s Goodness-of-Fit (GoF) test was used to determine whether the categorical variables followed a hypothesised distribution for all three models. Pearson χ^2^ values of *p* = 0.2560 for the first model, *p* = 0.3951 for the second, and *p* = 0.2655 for the third indicated a reasonably good fit for all three GEE models. A *p*-value of <0.05 was considered statistically significant for all tests. STATA, version 16.1, was used for all analyses.

## Results

A total of 302 CHS physicians participated in the study across eight training sessions. About 61% of study participants were male and more than half (56%) were aged 36 years or more. In terms of seniority, almost half of the study participants (49%) had been working in the CHSs for 11 years or more (Table 1).

**TABLE 1:** Background characteristics of study participants (Vietnam, 2019).

Characteristics		n	%
Gender	Male	184	60.9
	Female	118	39.1
Age group	≤35 years	133	44.0
	≥36 years	169	56.0
Mean of age ( $\bar{X}$ *± SD*) years		38.8 ± 8.2	
Seniority	≤10 years	153	50.7
	≥11 years	149	49.3
Mean of seniority ( $\bar{X}$ *± SD*) years		12.4 ± 8.3	
Province	Ha Giang	110	36.4
	Dien Bien	84	27.8
	Yen Bai	108	35.8

The proportions of CHS physicians providing correct answers regarding the diagnosis and treatment of mothers and children. Female physicians answered more pregnancy-related questions correctly than did male physicians. More than 76% of female physicians, but only 51% of males, provided correct answers on symptoms of preeclampsia, and this difference was statistically significant (*p* < 0.05). Similar results we observed for symptoms of ectopic pregnancy. Younger doctors demonstrated better knowledge of child care than their older counterparts. When asked about the diagnosis and management of dehydration in childhood diarrhoea, 50% of physicians aged less than 35 years answered correctly, and this figure dropped to 34% among doctors aged 36 years or more. When physicians were grouped according to their years of experience at the CHSs, differences were more modest for both pregnancy care and child care questions (Table 2).

**TABLE 2:** Proportions of Commune Health Station physicians providing correct answers regarding maternal and child health-related diagnosis and treatment (Vietnam, 2019).

Question	Correct answers provided										
	Overall n (%)		Sex (%)		Age (%)		Seniority (%)		Province (%)		
**Maternal Health (Pregnancy care)**			**Male**	**Female**	**<35 years**	**≥35 years**	**<10 years**	**≥10 years**	**Ha Giang**	**Dien Bien**	**Yen Bai**
Number of required antenatal check-ups	219	72.5	72.8	72.0	75.1	70.4	72.5	72.5	71.8	73.8	72.2
Routine antenatal check-ups structure	193	63.9	**50.7**	**72.0**	**71.4**	**56**	68.0	59.3	65.4	69.0	58.3
Symptom of ectopic pregnancy	182	60.3	**54.3**	**69.5**	61.6	59.1	58.8	61.7	52.7	61.9	66.7
Definition of gestational hypertension	131	43.4	39.7	49.1	42.9	43.8	42.5	44.3	40.9	38.1	50.0
Symptom of preeclampsia	198	65.6	**50.7**	**76.3**	68.4	63.3	64.0	67.1	61.8	64.3	70.4
Third trimester warning signs	208	68.9	65.8	73.7	73.7	65.1	70.6	67.1	**62.7**	**63.1**	**79.6**
**Child Health**											
Risk factors for acute diarrhoea	69	20.9	21.7	24.6	**28.6**	**18.3**	**27.4**	**18.1**	18.2	20.2	29.6
Diagnostic criteria of dehydration	124	40.1	**34.2**	**51.7**	**49.6**	**34.3**	43.8	38.2	37.3	38.1	47.2
Dehydration management practices	122	40.4	38.0	44.0	**48.9**	**33.7**	43.8	36.9	41.8	41.7	38.0
Diagnostic criteria for childhood pneumonia	58	19.2	**14.1**	**27.1**	21.0	17.7	20.9	17.4	**10.9**	**19.0**	**27.8**
Danger signs of childhood illness	122	40.4	36.4	46.6	46.6	35.5	43.8	36.9	37.3	34.5	48.1

Bold denoted the associations are significant at *p* < 0.05 (Pearson’s Chi-square).

Mean numbers of correct answers as well as comparisons of mean scores according to physicians’ characteristics. The mean number of correct answers across all participants was 5.4 out of 11. Proportionately, doctors correctly answered more questions related to pregnancy care (3.7 out of 6) compared to questions on child healthcare (1.6 out of 5). Female doctors’ overall score was 6.1 whereas the score for male doctors was 4.9 out of 11. Female doctors also performed better than their male counterparts on both pregnancy care (4.1 vs. 3.1) and child health (1.9 vs. 1.4), and the differences were statically significant (*p* < 0.01). Doctors younger than 35 years of age performed better on child health-related questions compared to doctors in the older age group (1.9 vs. 1.4, *p* = 0.0007). Likewise, the physicians who had been working in CHSs for less than 10 years performed better than the more experienced doctors on the child care questions (1.8 vs. 1.4, *p* < 0.05) (Table 3).

**TABLE 3:** Mean number of correct answers by doctor characteristics (Vietnam, 2019).

Physicians characteristics	Overall, mean number of correct answers		Mean number of correct answers on pregnancy care		Mean number of correct answers on child healthcare	
	Mean/11	*p*-Value	Mean/6	*p*-Value	Mean/5	*p*-Value
Overall	5.4	—	3.7		1.6	
Age						
<35 years	**5.9**		3.9		**1.9**	
≥35 years	**5.0**	**0.0097**	3.6	0.1307	**1.4**	**0.0007**
Gender						
Male	**4.9**		**3.5**		**1.4**	
Female	**6.1**	**0.0012**	**4.1**	**0.0052**	**1.9**	**0.0028**
Seniority						
<10 years	5.6		3.8		**1.8**	
≥10 years	5.2	0.2912	3.7	0.8564	**1.4**	**0.0477**
Province						
Ha Giang	5.0		3.5		1.5	
Dien Bien	5.2		3.7		1.5	
Yen Bai	5.8	0.7140	4.0	1.6896	1.9	0.1094

Bold values are significantly associated with doctors’ characteristics.

The crude and adjusted odds ratios (AOR) and demonstrates the association between physicians’ characteristics and answering more than half of the questions correctly. In the adjusted logistic regression, female physicians are 2.20 (95%CI: 1.35–3.59, *p* = 0.002) times more likely to answer correctly than their male counterparts. Similar odds were observed for the questions on pregnancy care (AOR 2.12, 95% CI: 1.30–3.43, *p* = 0.002) and child care (AOR 2.56, 95% CI: 1.50–4.36, *p* = 0.001). Physicians aged 35 years or more were significantly less likely to answer correctly (AOR 0.35, 95%CI: 0.15–0.81, *p* = 0.014 for all questions; AOR 0.34, 95% CI: 0.14–0.83, *p* = 0.019 for pregnancy care; and AOR 0.26, 95%CI: 0.10–0.70, *p* = 0.007 for child care). Physicians who had been working in CHSs >10 years were 2.80 times (95% CI: 1.16–6.72, *p* = 0.021) more likely to answer more than half of the pregnancy-related questions correctly than were the physicians having worked <10 years; this result is not surprising, as more female physicians have worked in CHSs >10 years. Physicians from Yen Bai province were more likely to correctly answer more than half of the questions on both maternal and child health compared to doctors from the other two provinces. In the adjusted model, doctors in Yen Bai province were 2.16 times (95% CI: 1.21–3.85, *p* = 0.009) more likely to answer correctly than the doctors from Ha Giang province (Table 4).

**TABLE 4:** Associations between physicians’ characteristics and answering more than half of questions correct (Vietnam, 2019).

	Overall, correct answer				Correct answers on pregnancy care				Correct answers on child healthcare			
Doctor characteristics	OR (95% CI)	*p*-Value	AOR (95% CI)	*p*-Value	OR (95% CI)	*p*-Value	AOR (95% CI)	*p*-Value	OR (95% CI)	*p*-Value	AOR (95% CI)	*p*-Value
Age												
<35 years	—		—		—		—		—		—	
≥35 years	0.75 (0.47–1.18)	0.215	**0.35** **(0.15–0.81)**	**0.014**	1.02 (0.64–1.61)	0.938	**0.34** **(0.14–0.83)**	**0.019**	**0.50** **(0.30–0.82)**	**0.007**	**0.26** **(0.10–0.70)**	**0.007**
Gender												
Male	—		—		—		—		—		—	
Female	**2.17** **(1.34–3.51)**	**0.001**	**2.20** **(1.35–3.59)**	**0.002**	**2.14** **(1.34–3.42)**	**0.002**	**2.12** **(1.30–3.43)**	**0.002**	**2.37** **(1.42–3.96)**	**0.001**	**2.56** **(1.50–4.36)**	**0.001**
Seniority												
<10 years	—		—		—		—		—		—	
≥10 years	1.03 (0.66–1.62)	0.891	1.83 (0.81–4.17)	0.147	1.49 (0.94–2.34)	0.087	**2.80** **(1.16–6.72)**	**0.021**	0.68 (0.41–1.12)	0.130	1.41 (0.64–2.55)	0.490
Province												
Ha Giang	—		—		—		—		—		—	
Dien Bien	1.13 (0.64–1.99)	0.678	1.13 (0.62–2.03)	0.689	1.10 (0.61–1.97)	0.746	1.14 (0.62–2.07)	0.676	1.35 (0.70–2.61)	0.371	1.28 (0.64–2.55)	0.490
Yen Bai	**1.90** **(1.11–3.27)**	**0.020**	**2.16** **(1.21–3.85)**	**0.009**	**2.10** **(1.22–3.61)**	**0.007**	**2.16** **(1.22–3.84)**	**0.008**	**1.94** **(1.07–3.55)**	**0.030**	**2.63** **(1.36–5.09)**	**0.004**

OR: odds ratios, AOR: Adjusted OR, CI: Confidence Interval. Bold denoted the associations are significant at *p* < 0.05.

## Discussion

Study findings suggest the need for sustained medical education in the areas of maternal and child health for primary care physicians in Vietnam. CME helps primary care physicians to keep their clinical knowledge up to date, which subsequently leads to improved patient care and health outcomes [21]. Most of the physicians who participated in the study were unable to answer all 11 questions correctly. Overall, physicians did not perform well in answering some critical primary care questions on childhood illness, providing justification for the requirement of HMU’s training program for CHS physicians in northern Vietnam. Compared to older physicians, young doctors performed better in answering questions on child care. When physicians were grouped according to sex, female physicians performed better on pregnancy care questions than did the male physicians. In terms of working experience, the physicians who were recently hired at CHSs or had less than 10 years of working experience had better knowledge of child care compared to the more experienced physicians.

Overall, physicians’ poor knowledge of child care is concerning. Only one-quarter of physicians correctly answered all questions on Integrated Management of Childhood Illness (IMCI) protocols, diagnosis and management of childhood diarrhoea, and identification of danger signs indicating the need for immediate referral of children to medical facilities. The IMCI has been considered a very important intervention for reducing under-five child mortality across many low-income countries. IMCI was initiated by the World Health Organization (WHO) and the United Nations Children’s Fund (UNICEF) in the mid-1990s to support healthcare providers in the diagnosis and treatment of children in under-resourced regions. As part of this initiative, primary care physicians in more than 113 countries, including Vietnam, were trained on IMCI guidelines. Despite these efforts, only 19% of CHS physicians who participated in our study correctly answered a question concerning diagnostic criteria for pneumonia in children aged 2–12 months. This is not surprising, as previous studies have reported non-adherence to IMCI guidelines by primary care physicians in many low- and middle-income countries [22–24]. There is substantial evidence suggesting that a single episode of physician training may not be sufficient to sustain knowledge and secure adherence to the IMCI guidelines [22, 24, 25]. Taken together, these data highlight the importance of ongoing IMCI training for CHS physicians, with special attention to the post-training follow-up and additional support of physicians with a practice manual of IMCI protocols [23].

As was the case for the IMCI guidelines, CHS physicians also performed poorly in answering questions on the diagnosis and management of dehydration in childhood diarrhoea. About 60% of physicians failed to provide correct answers related to this topic. Similar findings have been reported in other low-income settings. For example, a cross-sectional study conducted in Kolkata, India found that 43% of physicians possessed poor overall knowledge of diarrhoea diagnosis and treatment [26]. Accurate, early diagnosis of diarrhoeal dehydration by physicians in primary care settings is essential to prioritise the treatment of critically ill patients. Timely recognition of symptoms and proper assessment of the degree of dehydration enable stabilization and rapid implementation of rehydration strategies [27]. Despite recent progress in reducing mortality related to diarrhoeal diseases, the Lancet Global Burden of Disease series has suggested that diarrhoea was responsible for an estimated 5,33 ,768 deaths among children under-five globally in 2017, a rate of 78.4 deaths (70.1–87.1) per 1,00 ,000 children [28]. A study conducted in 2015 reported an estimated incidence of diarrhoea in children under five in rural Vietnam at 271 episodes/1,000 infant-years of observation [29], indicating a need for better physician training and additional resources to adequately diagnose and manage dehydration in childhood diarrhoea.

Physicians’ gender has been identified as an important factor to consider in providing CME in pregnancy care, as male doctors in our study were significantly less likely to provide correct answers on pregnancy-related issues compared to female physicians. There may be higher demand from patients seeking maternal or child care for female physicians, based on an expectation that they possess greater expertise and maintain enhanced knowledge related to women’s health [30]. A systematic review has also revealed that pregnant patients favoured female gynaecologist-obstetricians over males [31]. However, in Vietnam, most CHSs have only one physician, and the majority of them are male. Communities served by CHSs with male physicians deserve quality maternal healthcare. Thus, it is important for the Ministry of Health to provide continued training on maternal health for the male physicians in the CHSs. Our study revealed that only 39% of physicians in the CHSs are female. A study in India observed that the absence of female physicians in rural areas could compromise maternal healthcare and that the presence of female physicians in community health centres was associated with higher utilization of maternal health services [32]. These findings suggest that encouraging and incentivising more female physicians to practice in primary care settings may be an effective strategy to improve pregnancy-related outcomes in Vietnam.

Physician age and years of experience may be important determinants of the level of expertise in clinical practices related to maternal and child health. Intuitively, physicians’ experience, clinical skills and knowledge acquired over time in primary care settings would be expected to promote better healthcare and clinical outcomes. On the other hand, it is plausible that physicians’ knowledge may fall out of date as scientific advances lead to changes in recommended clinical practices. Our findings support the latter scenario, as physicians younger than 35 years or having worked in CHSs for less than 10 years scored better than their older or more experienced counterparts. A systematic review assessing the relationship between physician experience and clinical knowledge reported that older physicians possessed inferior clinical knowledge, demonstrated low adherence to treatment protocols and performed poorly on measures of proficiency in clinical care [33]. Therefore, it is important to consider targeting older physicians in developing CME curricula on maternal and child care.

### Limitations

Interpretation of our findings requires consideration of the study’s limitations. Our study participants were physicians employed in the CHSs of three Northern provinces, and findings may not be generalizable to all physicians working in CHSs across Vietnam. Given the cross-sectional design of this study, we were unable to determine the degree to which our results were attributable to changes in knowledge with physicians’ socio-demographic characteristics. The participants had a relatively short time (15 min) to complete the questionnaire of 48 items (including 11 items for maternal and child care), therefore differences in speed of recall may have affected test performance independently of physicians’ baseline knowledge. Although questionnaires were self-administered, supervision by faculty members during the completion of the test prevented participants from consulting with one another. Finally, this paper investigated knowledge of maternal and child care; further studies are required to evaluate the practical skills and clinical performance of physicians working at CHSs.

### Conclusion

Overall, our study has generated important findings related to physician knowledge of maternal and child care. The physicians who participated in this study possessed relatively poor knowledge of pregnancy care and diagnosis and management of childhood illnesses, including dehydration in diarrhoea. The results strongly support the requirement for continuing medical education to improve doctors’ proficiency in these areas. The significant disparities in knowledge according to physicians’ age, gender and seniority must be considered carefully in training activities. Adequate CME is expected to effectively address physicians’ knowledge gaps and help them provide high-quality pregnancy and child care throughout their careers.

## References

[1] Ministry of Health Viet Nam, Partnership for Maternal, Newborn and Child Health, WHO, World Bank and Alliance for Health Policy and Systems Research. Success Factors for Women’s and Children’s Health: Viet Nam. Geneva: World Health Organisation (2014). Available from: https://www.who.int/pmnch/knowledge/publications/vietnam_country_report.pdf (Accessed September 20, 2021).

[2] Målqvist M Hoa DT Thomsen S. Causes and Determinants of Inequity in Maternal and Child Health in Vietnam. BMC Public Health (2012) 12:641. 10.1186/1471-2458-12-641 22883138 PMC3534083

[3] McBride B Nguyen LT Wiljer D Vu NC Nguyen CK O'Neil J. Development of a Maternal, Newborn and Child mHealth Intervention in Thai Nguyen Province, Vietnam: Protocol for the mMom Project. JMIR Res Protoc (2018) 7(1):e6. 10.2196/resprot.7912 29326095 PMC5785686

[4] Lee HY Van Do D Choi S Trinh OT To KG. Trends and Determinants of Infant and Under-Five Childhood Mortality in Vietnam, 1986-2011. Glob Health Action (2016) 9:29312. 10.3402/gha.v9.29312 26950560 PMC4780095

[5] Duong DV Binns CW Lee AH. Utilization of Delivery Services at the Primary Health Care Level in Rural Vietnam. Soc Sci Med (2004) 59(12):2585–95. 10.1016/j.socscimed.2004.04.007 15474211

[6] General Statistics Office. Statistical Yearbook of Viet Nam 2017. Hanoi: Statistical Publishing House (2018).

[7] Vu LT Bales S Bredenkamp C. What Drives Utilization of Primary Care Facilities in Vietnam? Evidence From a Facility Survey. Washington, DC: The World Bank (2019). Available from: https://openknowledge.worldbank.org/bitstream/handle/10986/32186/What-Drives-Utilization-of-Primary-Care-Facilities-in-Vietnam-Evidence-from-a-Facility-Survey.pdf?sequence=1 (Accessed September 20, 2021).

[8] Kien VD Van Minh H Giang KB Nguyen V Ng N Tuan LT Views by Health Professionals on the Responsiveness of Commune Health Stations Regarding Non-Communicable Diseases in Urban Hanoi, Vietnam: A Qualitative Study. BMC Health Serv Res (2018) 18(1):392. Published 2018 May 31. 10.1186/s12913-018-3217-4 29855320 PMC5984436

[9] Hoa NT Derese A Peersman W Markuns JF Willems S Tam NM. Primary Care Quality in Vietnam: Perceptions and Opinions of Primary Care Physicians in Commune Health Centers–A Mixed-Methods Study. PLoS One (2020) 15(10):e0241311. 10.1371/journal.pone.0241311 33119666 PMC7595414

[10] Hoa NT Tam NM Derese A Markuns JF Peersman W. Patient Experiences of Primary Care Quality Amongst Different Types of Health Care Facilities in central Vietnam. BMC Health Serv Res (2019) 19(1):275. 10.1186/s12913-019-4089-y 31046750 PMC6498623

[11] Graner S Mogren I Krantz G Klingberg-Allvin M Duong le Q. Maternal Health Care Professionals' Perspectives on the Provision and Use of Antenatal and Delivery Care: A Qualitative Descriptive Study in Rural Vietnam. BMC Public Health (2010) 10:608. 10.1186/1471-2458-10-608 20946681 PMC3091560

[12] Huy NV Hieu TT Mai NT Thang NH Nga TT Nam NDH Human Resources for Commune Health Centers as Per National Standards: The Case of Vietnam. Fam Med Med Sci Res (2019) 8:236.

[13] Duong DB. Understanding the Service Availability for Non-Communicable Disease Prevention and Control at Public Primary Care Centers in Northern Vietnam, Doctoral Dissertation. Harvard Medical School (2015).

[14] Heo J Kim SY Yi J Yu SY Jung DE Lee S Maternal, Neonatal, and Child Health Systems Under Rapid Urbanization: A Qualitative Study in a Suburban District in Vietnam. BMC Health Serv Res (2020) 20:90. 10.1186/s12913-019-4874-7 32024537 PMC7003413

[15] Anh CT. Development of Vietnamese Medical Staff in the Period of Accelerated Industrialization and Modernization of the Country and International Integration. Asian J Soc Sci (2020) 5(1):39. 10.20849/ajsss.v5i1.729

[16] MOH. Niên Giám Thống Kê Y Tế Năm 2018 - Health Statistics Yearbook (2018). Available from: https://moh.gov.vn/thong-ke-y-te/-/asset_publisher/nEY3Q7enxRKG/content/nien-giam-thongke-y-te-nam-2018 (Accessed June 17, 2021).

[17] Van Minh H Do YK Bautista MA Tuan Anh T. Describing the Primary Care System Capacity for the Prevention and Management of Non‐communicable Diseases in Rural Vietnam. Int J Health Plann Manage (2014) 29(2):e159–73. 10.1002/hpm.2179 23553675

[18] Vujicic M Shengelia B Alfano M Thu HB. Physician Shortages in Rural Vietnam: Using a Labor Market Approach to Inform Policy. Soc Sci Med (2011) 73(7):970–7. 10.1016/j.socscimed.2011.06.010 21839563

[19] Thu NT Wilson A McDonald F. Motivation or Demotivation of Health Workers Providing Maternal Health Services in Rural Areas in Vietnam: Findings from a Mixed-Methods Study. Hum Resour Health (2015) 13(91):91–11. 10.1186/s12960-015-0092-5 26626015 PMC4667451

[20] Oanh TTM Phuong NK Tuan KA. Sustainability and Resilience in the Vietnamese Health System. Vietnam: Health Strategy and Policy Institute (2021). Available from: http://www3.weforum.org/docs/WEF_PHSSR_Vietnam_Report.pdf (Accessed June 18, 2021).

[21] Emami Z Kouhkan A Khajavi A Khamseh ME. Knowledge of Physicians Regarding the Management of Type Two Diabetes in a Primary Care Setting: The Impact of Online Continuous Medical Education. BMC Med Educ (2020) 20:374. 10.1186/s12909-020-02212-3 33081765 PMC7574317

[22] Nguyen DT Leung KK McIntyre L Ghali WA Sauve R. Does Integrated Management of Childhood Illness (IMCI) Training Improve the Skills of Health Workers? A Systematic Review and Meta-Analysis. PloS one (2013) 8(6):e66030. 10.1371/journal.pone.0066030 23776599 PMC3680429

[23] Abdo HA Kamel MH Seif N. Primary Care Physicians’ Adherence to IMCI Guidelines, Ismailia, Egypt. Asian J Pharm Nurs Med Sci (2016) 4(3).

[24] Lange S Mwisongo A Mæstad O. Why Don't Clinicians Adhere More Consistently to Guidelines for the Integrated Management of Childhood Illness (IMCI)? Soc Sci Med (2014) 104:56–63. 10.1016/j.socscimed.2013.12.020 24581062

[25] Horwood C Vermaak K Rollins N Haskins L Nkosi P Qazi S. An Evaluation of the Quality of IMCI Assessments Among IMCI Trained Health Workers in South Africa. PLoS One (2009) 4(6):e5937. 10.1371/journal.pone.0005937 19536288 PMC2693922

[26] Kanungo S Mahapatra T Bhaduri B Mahapatra S Chakraborty N Manna B Diarrhoea-Related Knowledge and Practice of Physicians in Urban Slums of Kolkata, India. Epidemiol Infect (2014) 142(2):314–26. 10.1017/S0950268813001076 23659645 PMC9151173

[27] Vega RM Avva U. Pediatric Dehydration. StatPearls (2020). 28613793

[28] Troeger CE Khalil IA Blacker BF Biehl MH Albertson SB Zimsen SR Quantifying Risks and Interventions that Have Affected the burden of Diarrhoea Among Children Younger Than 5 Years: An Analysis of the Global Burden of Disease Study 2017. Lancet Infect Dis (2020) 20(1):37–59. 10.1016/S1473-3099(19)30401-3 31678029 PMC7340495

[29] Anders KL Thompson CN Thuy NT Nguyet NM Tu le TP Dung TT The Epidemiology and Aetiology of Diarrhoeal Disease in Infancy in Southern Vietnam: A Birth Cohort Study. Int J Infect Dis (2015) 35:3–10. 10.1016/j.ijid.2015.03.013 25813553 PMC4508461

[30] Lahat A Assouline-Dayan Y Katz LH Fidder HH. The Preference for an Endoscopist Specific Sex: A Link between Ethnic Origin, Religious Belief, Socioeconomic Status, and Procedure Type. Patient Prefer Adherence (2013) 7:897–903. 10.2147/PPA.S48468 24043933 PMC3772755

[31] Janssen SM Lagro-Janssen AL. Physician's Gender, Communication Style, Patient Preferences and Patient Satisfaction in Gynecology and Obstetrics: A Systematic Review. Patient Educ Couns (2012) 89(2):221–6. 10.1016/j.pec.2012.06.034 22819711

[32] Bhan N McDougal L Singh A Atmavilas Y Raj A. Access to Women Physicians and Uptake of Reproductive, Maternal and Child Health Services in India. EClinicalMedicine (2020) 20:100309. 10.1016/j.eclinm.2020.100309 32300752 PMC7152807

[33] Choudhry NK Fletcher RH Soumerai SB. Systematic Review: The Relationship Between Clinical Experience and Quality of Health Care. Ann Intern Med (2005) 357:260–73. 10.7326/0003-4819-142-4-200502150-00008 15710959

