# Supplementary material for: Knowledge of Medical Education on Maternal and Child Primary-Care Among Physicians: A Cross-Sectional Study
Source: Int J Public Health. 2024 Jul 4;69:1606536. doi: 10.3389/ijph.2024.1606536 (PMC11254614; doi:10.3389/ijph.2024.1606536)
Supplement: Supplementary file 1 [file Table1.DOCX]

**Supplementary Table1: Question-items, original questions, response options and correct answer(s) in the questionnaire on maternal and child care**

| **Question-items** | **Original questions** | **Response options** | **Correct answer(s)** |
| --- | --- | --- | --- |
| **Pregnancy care** | | | |
| Number of required antenatal check-ups | What is the minimum number of antenatal check-ups required by Ministry of Health? | A. 3 times  B. 4 times  C. 5 times  D. When there are abnormal symptoms | B. 4 times |
| Routine antenatal check-ups structure | Please fill in the blanks about your knowledge of the topics to be covered at each antenatal check-ups. | Open ended question to be completed using key words, examples below:  - Nutrition  -Take vitamin/mineral supplements  - Tetanus vaccine during pregnancy  - Personal hygiene  - Routine physical activities  -Signs of danger during pregnancy (pain, bleeding)  - Make an appointment for a follow-up appointment | At least 4 key words |
| Symptom of ectopic pregnancy | What symptoms suggest an ectopic pregnancy? | A. Lower abdominal pain, urinary retention, nausea  B. Missed period, nausea, chest tightness  C. Late period, lower abdominal pain, abnormal vaginal bleeding  D. Abdominal pain, vomiting, menorrhagia | C. Late period, lower abdominal pain, abnormal vaginal bleeding |
| Definition of gestational hypertension | What is the definition of gestational hypertension? | A. Hypertension occurs after the 20th week of pregnancy and returns to normal 6 weeks after delivery.  B. …… after the 12nd week  C. …. Before the 16th week  D. Any time during pregnancy | A. Hypertension occurs after the 20th week of pregnancy and returns to normal 6 weeks after delivery. |
| Symptom of preeclampsia | Identify three important symptoms of pre-eclampsia? | A. Hypertension, increase in liver enzymes, decrease in blood proteins  B. Increased blood pressure, increased liver enzymes, decreased platelets  C. Hypertension, edema, excess proteinuria  D. Hypertension, edema, decreased proteinuria | C. Hypertension, edema, excess proteinuria |
| Third trimester warning signs | What are the third trimester warning signs? | A. The fetus has reduced movement  B. Frequent abdominal pain  C. Rapid weight gain  D. All of the above signs | D. All of the above signs |
| **Child care** | | | |
| Risk factors for acute diarrhoea | Which of the following practices increase the risk of acute diarrhoea? | A. Babies are fed with breast milk and formula milk at the same time  B. Leaving prepared milk/ cooked food at room temperature for a long time  C. The child is only fed with formula milk  D. All of the above | B. Leaving prepared milk/ cooked food at room temperature for a long time |
| Diagnostic criteria of dehydration | What are the diagnostic criteria for dehydration in acute diarrhoea? | A. Sunken eyes  B. Skin pinch disappears slowly  C. Unable to drink water or drinking poorly  D. All of the above signs | D. All of the above |
| Dehydration management practices | Managing dehydration in diarrhoea children | A. Assess the degree of dehydration  B. Rehydration, electrolytes according to the degree of dehydration  C. Limit the child's food and drink to reduce the number of diarrhoea episodes so that the illness can be cured quickly  D. Early antibiotics are needed to treat diarrhoea | A. Assess the degree of dehydration  B. Rehydration, electrolytes according to the degree of dehydration |
| Diagnostic criteria for childhood pneumonia | According to IMCI, what are the criteria of diagnosis of pneumonia on a child from 2-12 months of age? | A. Cough, fever, breathing rate of 60 times/minute or more  B. Cough, fever, breathing rate of 50 breaths/minute or more  c. Cough, fever, breathing rate of 40 times/minute or more  D. Cough, fever, breathing rate of 35 breaths/minute or more | B. Cough, fever, breathing rate of 50 breaths/minute or more |
| Danger signs of childhood illness | What are the danger signs indicating need to take sick children to see a medical facility immediately? | A. Lethargy, sleepiness is hard to wake up  B. Irritability  C. Noisy breathing while lying still  D. Fever 38.5 | A. Lethargy, sleepiness  C. Noisy breathing while lying still |
